# Hypoxia-induced ANGPTL4 sustains tumour growth and anoikis resistance through different mechanisms in scirrhous gastric cancer cell lines

**DOI:** 10.1038/s41598-017-11769-x

**Published:** 2017-09-11

**Authors:** Koichi Baba, Yoshihiko Kitajima, Shuusuke Miyake, Jun Nakamura, Kota Wakiyama, Hirofumi Sato, Keiichiro Okuyama, Hiroshi Kitagawa, Tomokazu Tanaka, Masatsugu Hiraki, Kazuyoshi Yanagihara, Hirokazu Noshiro

**Affiliations:** 1 Department of Surgery, Saga University Faculty of Medicine, 5-1-1, Nabeshima, Saga-shi, Saga, 849-8501 Japan; 2 Department of Surgery, National Hospital Organization Higashisaga Hospital, 7324, Ooaza Harakoga, Miyaki-cho, Miyaki-gun, Saga, 849-0101 Japan; 3 Department of Surgery, Saga-ken Medical Centre Koseikan, 400, Ooaza Nakahara, Kase-machi, Saga-shi, Saga, 840-8571 Japan; 4 Division of Translational Research, Exploratory Oncology Research & Clinical Trial Center, National Cancer Center, 6-5-1 Kashiwanoha, Kashiwa-shi, Chiba, 277-8577 Japan

## Abstract

Patients with scirrhous gastric cancer (SGC) frequently develop peritoneal dissemination, which leads to poor prognosis. The secreted protein angiopoietin-like-4 (ANGPTL4), which is induced by hypoxia, exerts diverse effects on cancer progression. Here, we aimed to determine the biological function of ANGPTL4 in SGC cells under hypoxia. ANGPTL4 levels were higher in SGC cells under hypoxia than in other types of gastric cancer cells. Hypoxia-induced *ANGPTL4* mRNA expression was regulated by hypoxia-inducible factor-1α (HIF-1α). Under hypoxic conditions, monolayer cultures of ANGPTL4 knockdown (KD) 58As9 SGC (58As9-KD) cells were arrested in the G_1_ phase of the cell cycle through downregulation of c-Myc and upregulation of p27, in contrast to control 58As9-SC cells. Moreover, the ability of 58As9-KD xenografts to form tumours in nude mice was strongly suppressed. When 58As9-KD cells were cultured in suspension, hypoxia strongly increased their susceptibility to anoikis through suppression of the FAK/Src/PI3K-Akt/ERK pro-survival pathway, followed by activation of the apoptotic factors caspases-3, -8 and -9. The development of peritoneal dissemination by 58As9-KD cells was completely inhibited compared with that by 58As9-SC cells. In conclusion, *ANGPTL4* is uniquely induced by hypoxia in cultured SGC cells and is essential for tumour growth and resistance to anoikis through different mechanisms.

## Introduction

Scirrhous gastric carcinoma (SGC) exhibits unique characteristics compared with other gastric carcinomas (GCs). Poorly differentiated adenocarcinoma or signet-ring cell carcinoma infiltrates diffusely in most patients with SGC, which is associated with worse prognosis than that of other GCs^1–3^. SGC invades rapidly and progressively, and cancer cells frequently seed the peritoneum, which accumulates ascites caused by peritoneal carcinomatosis^2, 3^. Even when curative surgery is applied, the survival rate of patients with SGC is extremely poor^2, 3^. Moreover, chemotherapy, radiotherapy and immunotherapy are insufficient to improve prognosis^3^. Therefore, the identification and isolation of specific molecules critical for SGC progression may be valuable by providing a better understanding of molecular pathogenesis. Such molecules may also serve as targets for therapy.

Hypoxia is a hallmark of solid tumour formation and an independent prognostic factor for malignant tumours^4, 5^. Adaptation to hypoxia is centrally mediated by the hypoxia-inducible factors (HIF)-1 and HIF-2^6–8^. HIFs enhance malignant phenotypes such as angiogenesis, invasion, metastasis and drug resistance^7, 8^. In GC, clinical and experimental evidence supports a pivotal function of HIFs that define the malignant phenotype^9–13^. Recently, a notable study of tumour hypoxia *in vivo* employed prostate-cancer xenografts expressing an EGFP reporter expressed under the control of the hypoxia-responsive element (HRE)^14^. The results revealed that orthotopic primary xenografts and xenograft-derived metastatic cells in the lymph node and peritoneum are hypoxic^14^. This study inspired our hypothesis that HIFs target genes that may contribute to the progression of primary and metastatic tumours.

Angiopoietin-like 4 (ANGPTL4) is a secreted member of the angiopoietin-like protein family (ANGPTL1–7), although its receptor has not been identified^15, 16^. Native full-length ANGPTL4 (F-ANGPTL4) can undergo proteolytic processing to generate an N-terminal coiled-coiled fragment (N-ANGPTL4) and a C-terminal fibrinogen-like domain (C-ANGPTL4)^15, 16^, although the function of ANGPTL4 is not fully defined. F-ANGPTL4 inhibits endothelial cell migration^15, 17^, and N-ANGPTL4 plays an endocrine regulatory role in lipid metabolism and insulin sensitivity^15, 18^. In contrast, C-ANGPTL4 regulates cancer growth, angiogenesis and metastasis^15, 16, 19^. However, the biological effects of ANGPTL4 on cancer cells are controversial^15, 16^. One study suggested critical roles for ANGPTL4 in the progression of GC^20^, although another reported conflicting data^21^.

The aim of the present study is thus to investigate the biological role of hypoxia-induced ANGPTL4 in SGC progression. We determined that ANGPTL4 expression was specifically induced by hypoxia in SGC cell lines, and we used siRNA knockdown (KD) techniques to evaluate the role of ANGPTL4 in cell cycle progression and resistance to anoikis in SGC cells cultured under hypoxic conditions.

## Results

### Analysis of ANGPTL4 expression in GC cell lines cultured under normoxia and hypoxia

**Figure 1:**
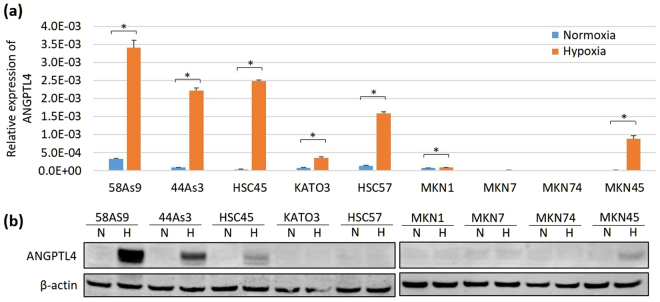
Analysis of ANGPTL4 expression in nine gastric cancer (GC) cell lines cultured under normoxia and hypoxia for 24 h. (**a**) RT-qPCR analysis of ANGPTL4 expression in nine GC cell lines. Relative expression of *ANGPTL4* mRNA was determined as the expression ratio of *ANGPTL4* mRNA/*β-actin* mRNA. The experiments were performed in triplicate and repeated three times. The data are presented as the mean ± SD. P values < 0.05 indicate a significant difference, as marked by an asterisk. (**b**) Western blot (WB) analysis of ANGPTL4 expression in nine GC cell lines. Strong ANGPTL4 expression (upper panel) was induced in 58As9 and 44As3 cells after 24 h of hypoxia. The internal marker β-actin is shown in the lower panel. The experiments were repeated three times. N, normoxia; H, hypoxia.

The expression of ANGPTL4 mRNA and protein was investigated in GC cell lines cultured under normoxic and hypoxic conditions. GC cell lines expressed little *ANGPTL4* mRNA and protein under normoxia (Fig. 1a,b). Under hypoxia, *ANGPTL4* mRNA levels were significantly elevated in the undifferentiated GC cells 58As9, 44As3, HSC45, HSC57, KATO3 and MKN45 compared with those of the differentiated GC cells MKN1, MKN7 and MKN74 (Fig. 1a). Western blot (WB) analysis showed hypoxic induction of ANGPTL4 in the undifferentiated GC cells 58As9, 44As3, HSC45 and MKN45 (Fig. 1b). Among the four GC cell lines, 58As9, 44As3 and HSC45 were derived from signet-ring cell carcinomas present in ascites or pleural effusion of different SGC patients. In particular, analyses showed that 58As9 and 44As3 SGC cells strongly expressed ANGPTL4 under hypoxia, so they were used in subsequent experiments.

### Regulation of ANGPTL4 mRNA levels by HIF-1α under hypoxia

**Figure 2:**
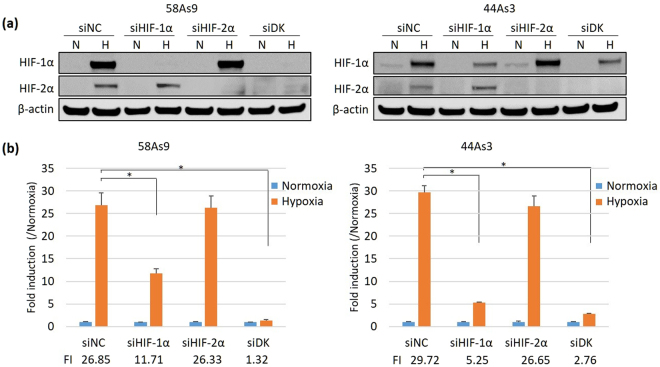
Analysis of the effects of HIF-α knockdown (KD) on ANGPTL4 expression in 58As9 and 44As3 SGC cells. (**a**) WB analysis of lysates prepared from cells transfected with siRNAs specific for HIF-1α or HIF-2α, or with both siRNAs (siDK). HIF-1α (upper panel), HIF-2α (middle panel). Control siRNA transfection is presented as siNegative control (siNC), and β-actin expression is shown in the lower panel. The experiments were repeated three times. (**b**) RT-qPCR analysis of *ANGPTL4* mRNA expression in 58As9 and 44As3 cells transfected with the HIF-α siRNAs under normoxia or hypoxia. Relative expression of *ANGPTL4* mRNA was determined as the expression ratio of *ANGPTL4* mRNA/*β-actin* mRNA. The expression of hypoxia/normoxia was further calculated for each transfectant and is presented as fold induction (FI). The experiments were performed in triplicate and repeated three times, and the data are presented as mean ± SD. P values ≤ 0.05 indicate a significant difference, as marked by an asterisk. siNC, negative control siRNA; siDK, double HIF-α siRNAs; N, normoxia; H, hypoxia.

To determine whether hypoxia-induced ANGPTL4 was regulated by HIF-α, HIF-1α and HIF-2α, we analysed the effects of HIF-α KD on ANGPTL4 mRNA expression in 58As9 and 44As3 SGC cells (Fig. 2a). WB analysis showed that HIF-1α or HIF-2α siRNAs effectively reduced the cognate HIF-α levels under hypoxia (Fig. 2a). RT-qPCR analysis demonstrated that hypoxic induction of *ANGPTL4* mRNA, which was assessed by determining the fold induction (FI) of hypoxia/normoxia, was significantly decreased in siHIF-1α KD cells compared with that for the negative control siRNA (siNC) (Fig. 2b). In contrast, the hypoxic induction of *ANGPTL4* mRNA was not influenced by siHIF-2α KD. Further, siDouble-Knockdown (siDK) transfection significantly inhibited *ANGPTL4* mRNA expression under hypoxia (Fig. 2b).

### Inhibition of ANGPTL4 expression in 58As9 and 44As3 KD cells

**Figure 3:**
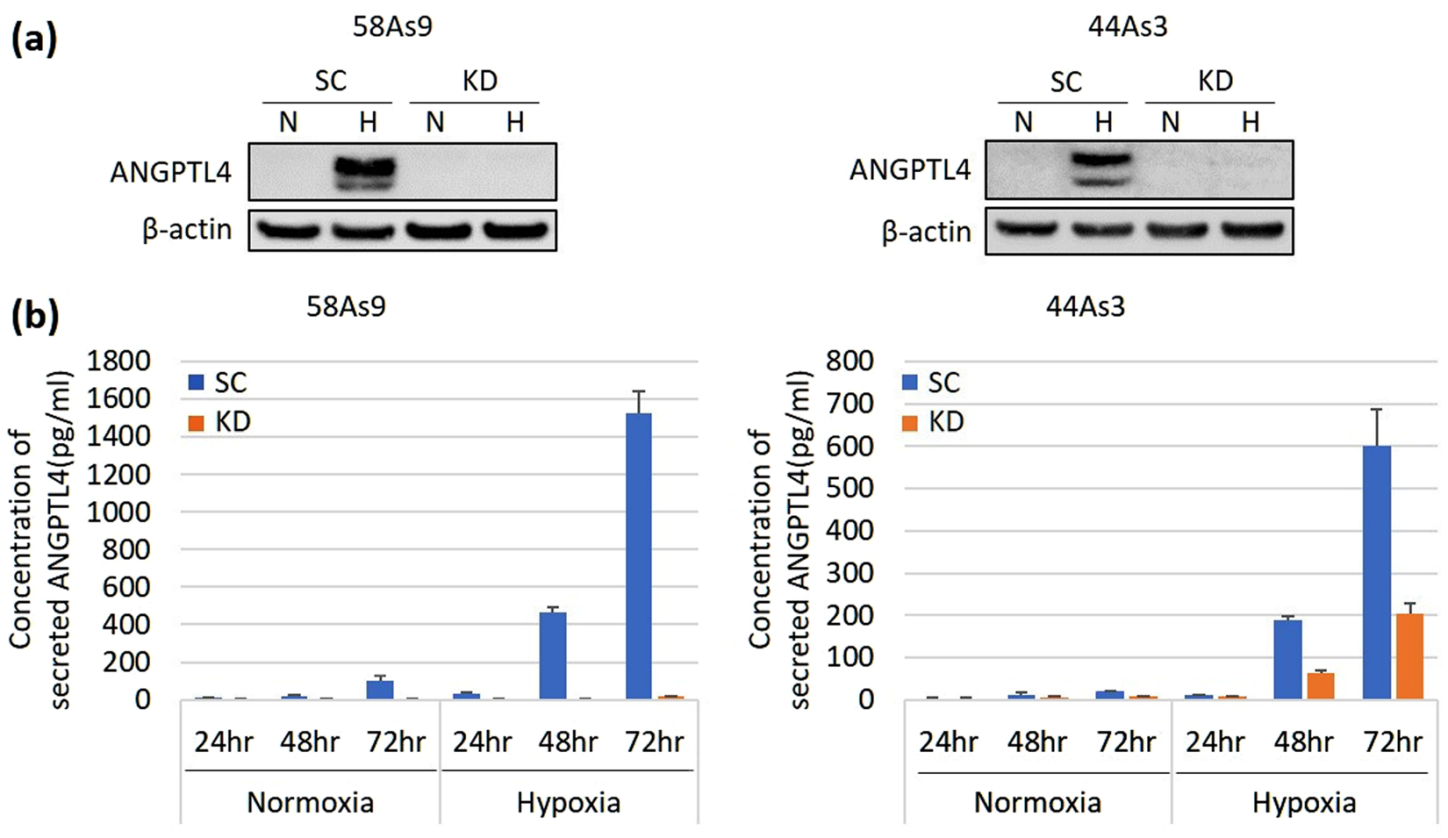
Establishment of stable ANGPTL4-KD clones of 58As9 and 44As3 cells. (**a**) WB analysis of the effect of ANGPTL4 KD on the 58As9 or 44As3 transfectant (upper panel). SC: negative scramble siRNA transfectant, KD: ANGPTL4 siRNA transfectant. The expression of internal control β-actin is shown in the lower panel. The experiments were repeated three times. (**b**) The concentration of secreted ANGPTL4 was estimated using an ELISA (58As9, left; 44As3, right). The experiments were performed in triplicate and repeated three times. The data are presented as the mean ± SD.

First, inhibition of ANGPTL4 expression in 58As9-KD and 44As3-KD cells was evaluated using WB analysis (Fig. 3a). ANGPTL4 expression was induced under hypoxia in 58As9-SC and 44As3-SC cells (Fig. 3a). However, the hypoxia-induced expression of ANGPTL4 was strongly inhibited in the cognate KD cells (Fig. 3a). Thereafter, an ELISA revealed that the levels of secreted ANGPTL4 increased with time under hypoxia in 58As9-SC and 44As3-SC cells, whereas the amount of secreted ANGPTL4 under normoxia was much smaller (Fig. 3b). In contrast, ANGPTL4 secretion was inhibited in 58As9-KD and 44As3-KD cells under hypoxia compared with that in the cognate SC cells (Fig. 3b). In particular, ANGPTL4 secretion was more strongly inhibited in 58As9-KD cells than that in 44As3-KD cells under hypoxia (Fig. 3b).

### *In vitro* effects of ANGPTL4 KD on the proliferation of 58As9 SGC cells

**Figure 4:**
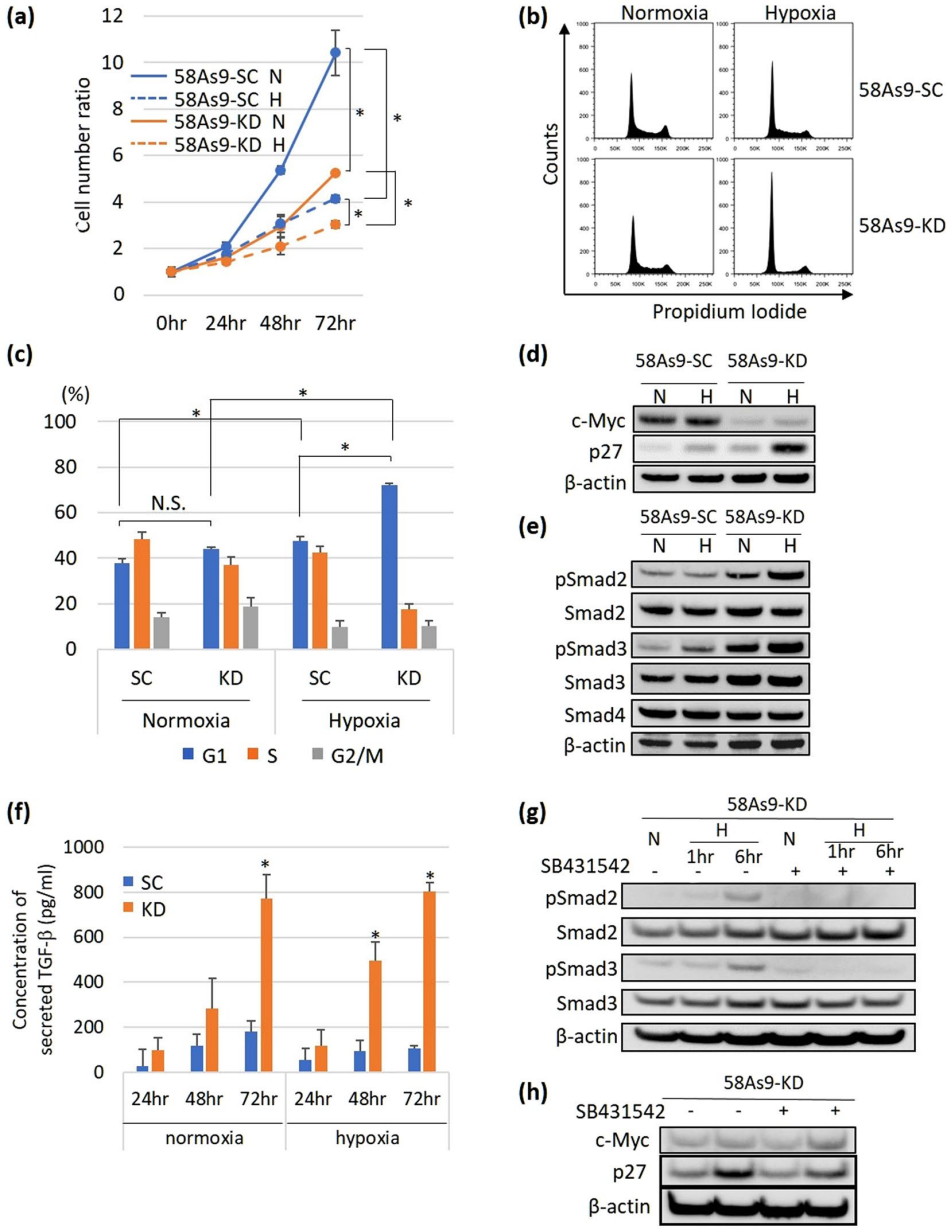
Cell proliferation and cell cycle analyses of 58As9 transfectants under normoxia and hypoxia. (**a**) Monolayer cultures of 58As9-SC (SC) and 58As9-KD (KD). The cell number ratio was estimated by dividing the cell number at 24, 48 or 72 h by the number at 0 h. The significance of the difference in the cell number ratios at 72 h between SC and KD cells was evaluated under normoxia or hypoxia. The difference in the cell number ratios was also assessed between normoxia and hypoxia in each of the SC and KD cells. P values ≤ 0.05 indicate a significant difference, as marked by an asterisk. (**b**) 58As9-SC and 58As9-KD cells were cultured for 24 h under normoxia and hypoxia and the cell cycle was analysed using flow cytometry. Distribution of cell number as determined by PI staining is shown. (**c**) Proportion of cells in the G_1_, S or G_2_/M phase of the cell cycle in 58As9-SC (SC) and 58As9-KD (KD) cells under normoxia and hypoxia. P values ≤ 0.05 indicate a significant difference, as marked by an asterisk. (**d**) WB analysis of c-Myc expression (upper panel), p27 (middle panel) and β-actin (lower panel) in 58As9-SC and 58As9-KD cells. N: normoxia, H: hypoxia. (**e**) WB analysis of the expression of TGF-β/Smad signalling pathway components in 58As9-SC and 58As9-KD cells. pSmad2: phosphorylated Smad2, pSmad3: phosphorylated Smad3. N: normoxia, H: hypoxia. (**f**) ELISA of TGF-β secreted from 58As9-SC (SC) and 58As9-KD (KD) cells. The TGF-β concentration (pg/ml) under normoxia and hypoxia was determined at the indicated times. Statistical comparison of the TGF-β concentration between normoxia and hypoxia was performed. P values ≤ 0.05 indicate a significant difference, as marked by an asterisk. (**g**) WB analysis of pSmad2/3 expression after treatment with 5 μg of the TGF-β inhibitor SB431542 in 58As9-KD cells. Expression of Smad2/3 and pSmad2/3 was analysed in 58As9-KD cells under normoxia (N) and hypoxia (H) in the presence or absence of SB431542 (5 µM) for 1 and 6 hr. (**h**) WB analysis of c-Myc and p27 in 58As9-KD cells, treated with or without SB431542 (5 µM) under normoxia (N) or hypoxia (H) for 24 hr. The experiments were performed in triplicate and repeated three times.

The ratio of the numbers of cells in monolayers of 58As9-SC or 58As9-KD cells decreased under hypoxia compared with that under normoxia (Fig. 4a). Compared with 58As9-SC cells, the ratio of 58As9-KD cells under normoxia or hypoxia significantly decreased (Fig. 4a).

The effects of *ANGPTL4* KD on the cell cycle were investigated by flow cytometry (Fig. 4b). The results revealed that the proportion of 58As9-SC cells in G_1_ phase was significantly increased in hypoxia (47.5%) compared with that in normoxia (37.7%) (Fig. 4c). Moreover, hypoxia dramatically elevated the percentage of 58As9-KD cells in G_1_ phase (72.0%) compared with normoxia (44.1%) (Fig. 4c). Under hypoxia, the population of 58As9-KD cells in G_1_ phase was significantly higher than that of 58As9-SC cells (Fig. 4c).

To further investigate the mechanism of the induction of cell cycle arrest in hypoxic 58As9-KD cells, we used WB analysis to measure the expression of cell cycle-related proteins. The expression of c-Myc was strongly inhibited in 58As9-KD cells compared with that in 58As9-SC cells under normoxia and hypoxia (Fig. 4d). Moreover, the levels of the cell cycle inhibitor p27 were conversely increased in 58As9-KD cells and were higher under hypoxia than under normoxia (Fig. 4d). The expression of other inhibitors such as p15, p16, p19 and p21 was not increased in hypoxic 58As9-KD cells compared with that in 58As9-SC cells (data not shown).

Our findings that c-Myc expression was downregulated and p27 was induced in hypoxic 58As9-KD cells focused our attention on TGF-β/Smad signalling (Fig. 4e). The levels of the phosphorylated forms of Smad2 (pSmad2) and Smad3 (pSmad3) were upregulated in 58As9-KD cells compared with those in 58As9-SC cells, and higher levels of pSmad2/pSmad3 were detected in hypoxic than in normoxic cells (Fig. 4e). An ELISA revealed that TGF-β was more highly secreted with time in 58As9-KD cells than in 58As9-SC cells under normoxia and hypoxia (Fig. 4f). Treatment with the TGF-β inhibitor SB431542 (5 μM) also completely suppressed Smad2/3 phosphorylation in 58As9-KD cells (Fig. 4g). SB431542 treatment increased c-Myc and reduced p27 expression in 58As9-KD cells under 24 h of hypoxia, compared with their levels with no treatment (Fig. 4h). However, SB431542 (5 μM) decreased the proliferation of 58As9-KD cells under normoxia (Fig. S1). Furthermore, the treatment did not affect cell proliferation or the cell cycle of the KD cells under hypoxia (Fig. S1).

### Effect of ANGPTL4 KD on the tumorigenicity of GC cell lines in nude mice

**Figure 5:**
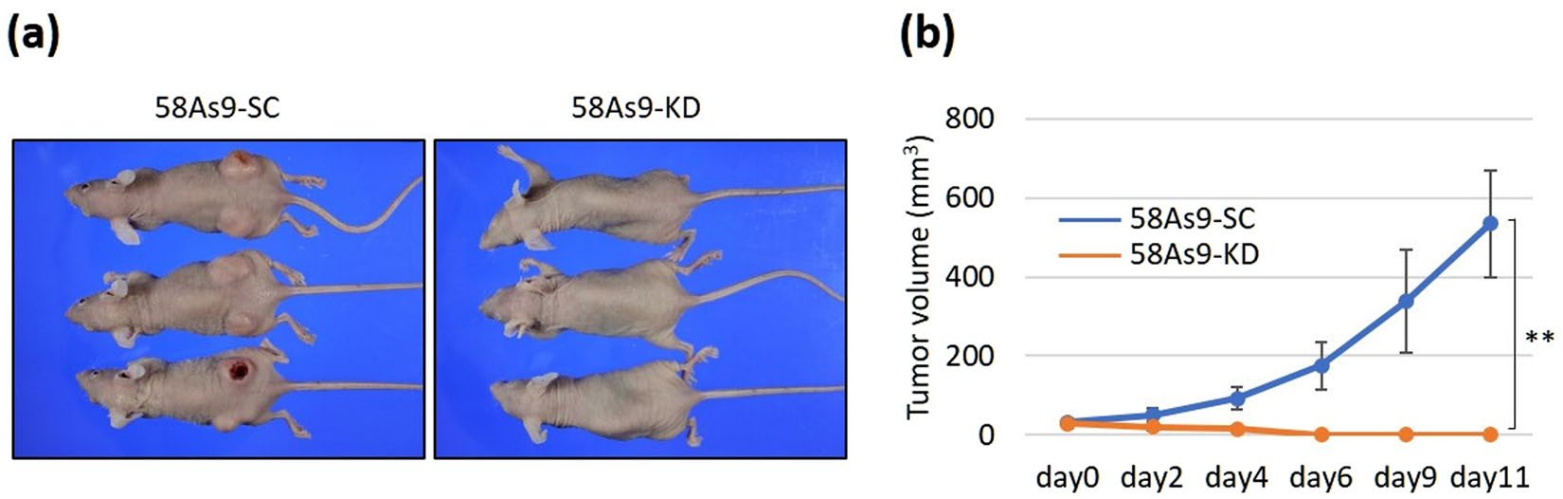
Analysis of the effect of ANGPTL4 KD on the growth of tumours in nude mice. (**a**) Image of nude mice implanted with 58As-SC or 58As9-KD cells. (**b**) Mean tumour size was estimated on the indicated days. Comparison of tumour size on day 11 after injection of mice injected with 58As-SC or 58As9-KD cells. Six tumours per three mice were analysed. The data are presented as the mean ± SD. P values ≤ 0.05 indicate a significant difference (**P ≤ 0.01). N, normoxia; H, hypoxia.

The ability of 58As9-KD xenografts to form tumours was significantly diminished compared with that of 58As9-SC cells (Fig. 5a). For example, 11 days after implantation, the volumes of tumours formed by 58As9-SC cells were significantly larger than those of tumours formed by 58As9-KD cells (Fig. 5b).

### *In vitro* effects of inhibiting ANGPLTL4 expression on anoikis in 58As9 cells and the effects of recombinant ANGPTL4 peptides on anoikis

**Figure 6:**
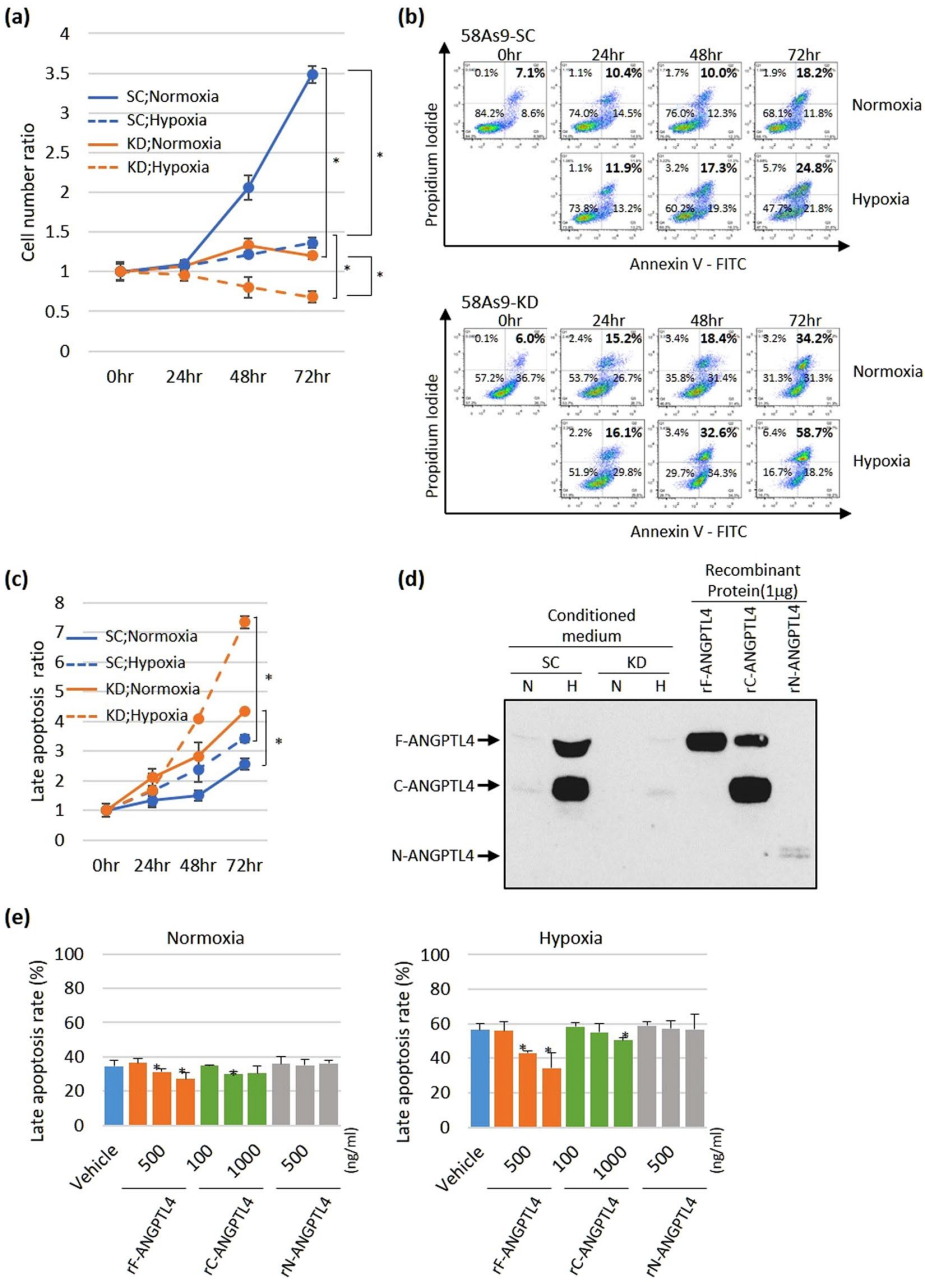
Analysis of the effect of ANGPTL4 on anoikis. (**a**) 58As9-SC and 58As9-KD cells were cultured in suspension, and the cell number ratio was estimated. The cell number ratios of 58As9-SC (SC) and 58As9-KD (KD) cells at 72 h under normoxia or hypoxia were compared. The cell number ratio at 72 h was also compared between normoxia and hypoxia in each of SC and KD cells. (**b**) Flow cytometric analysis of apoptosis. Distribution of cell number according to Annexin V and PI staining. The proportions of apoptotic or nonapoptotic 58As9-SC and 58As9-KD cells that were cultured as a suspension for 24–72 h are indicated in each panel. Detection of Annexin V^+^/PI^−^ (early apoptosis) or Annexin V^+^/PI^+^ (late apoptosis) was considered to indicate apoptosis, and values (bold) denote the rate of late apoptotic cells (%). The cell population at late apoptosis was more abundant in 58As9-KD than in 58As9-SC cells over time. (**c**) The late apoptosis ratios of 58As9-SC and 58As9-KD cells incubated under normoxia and hypoxia for 24–72 h were determined by estimating the ratio of the late apoptotic cell number at 72 h divided by the total cell number at 0 h. (**d**) WB analysis of F-, N- and C-ANGPTL4 expression in culture supernatants. In parallel, three forms of recombinant (r) peptides (1 μg) were subjected to WB. Bands corresponding to rF-ANGPTL4 and rC-ANGPTL4 were clearly observed, whereas the rN-ANGPTL4 band was weakly detected by this ANGPTL4 antibody. The mobility of the rC-ANGPTL4 band indicates that it formed a dimer. (**e**) The inhibitory effect of three rAPGPTL4 peptides on the anoikis of 58As9-KD cells. The 58As9-KD cells were treated with the recombinant forms of ANGPTL4 peptides at the indicated concentrations (ng/ml). The late apoptotic rates of cells treated with the vehicle or rANGPTL4 peptides were estimated by flow cytometric study. The experiments were performed in triplicate and repeated three times. The data are presented as the mean ± SD. P values ≤ 0.05 indicate a significant difference, as marked with an asterisk. N, normoxia; H, hypoxia; rF-ANGPTL4, full-length rANGPTL4; rC-ANGPTL4, C-terminal rANGPTL4; rN-ANGPTL4, N-terminal rANGPTL4.

We next investigated whether ANGPTL4 expression was critical for resisting anoikis. To evaluate anoikis *in vitro*, a suspension culture system was used. The cell number ratio of 58As9-SC cells increased with time to a greater extent under normoxia; however, the time-dependent elevation of cell number ratio was significantly suppressed under hypoxic conditions (Fig. 6a). In contrast, the ratio was strongly decreased in 58As9-KD cells compared with that of 58As9-SC cells under normoxia or hypoxia (Fig. 6a). The cell number ratio of 58As9-KD cells after 72 h was significantly lower than that of 58As9-SC cells under normoxia. Furthermore, the ratio of 58As9-KD cells decreased with time under hypoxia (Fig. 6a) and was significantly lower than that of 58As9-SC cells under hypoxia (72 h) (Fig. 6a).

We next investigated whether the differences in the cell number ratio were associated with apoptosis. A flow cytometric study showed that the apoptotic rate of 58As9-SC cells, estimated as the sum of Annexin V (−)/PI (+) and Annexin V (+)/PI (+), was increased with time to a greater extent under hypoxia than under normoxia (Fig. 6b). Apoptosis was also more strongly induced in 58As9-KD cells than in 58As9-SC cells under normoxia or hypoxia (Fig. 6b). Moreover, after culture for 72 h under normoxia or hypoxia, the late apoptosis ratio of 58As9-KD cells was significantly higher than that of 58As9-SC cells (Fig. 6c).

To investigate whether secreted ANGPTL4 regulated resistance to anoikis, 58As9 KD cells were treated with recombinant (r) ANGPTL4, and the apoptotic rate was analysed using flow cytometry under normoxia or hypoxia for 72 h. The culture supernatant from 58As9-SC and 58As9-KD cells was first subjected to WB analysis to evaluate the levels of secreted ANGPTL4 (Fig. 6d). F- and C-ANGPTL4 were induced and detected in the supernatant of a culture of hypoxic 58As9-SC cells and migrated to positions consistent with the molecular weight of rF-ANGPTL4 and rC-ANPTL4, respectively (Fig. 6d). N-ANGPTL4 was not secreted from hypoxic 58As9-SC cells, although the antibody exhibited lower affinity for rN-ANGPTL4 than that of rC-ANGPTL4 (Fig. 6d). These findings were confirmed by WB analysis using a different ANGPTL4 antibody (Ab2) with a higher affinity to rN-ANGPTL4 than rC-ANGPTL4 (Fig. S2). Thereafter, flow cytometric analysis demonstrated that treatment with rF-ANGPTL4 and rC-ANGPTL4 decreased the apoptotic rate of 58As9-KD cells under normoxia and hypoxia in a concentration-dependent manner, while treatment with rN-ANGPTL4 did not affect apoptosis (Fig. 6e). Treatment of 58As9-KD cells under normoxia or hypoxia with rF-ANGPTL4 (500 and 1000 ng/ml) significantly decreased apoptosis (Fig. 6e). Treatment with rC-ANGPTL4 (500 and 1000 ng/ml) induced a significant decrease in the number of apoptotic cells under normoxia or hypoxia (Fig. 6e).

### Regulation of the pro-survival signalling and apoptosis pathways by ANGPTL4

**Figure 7:**
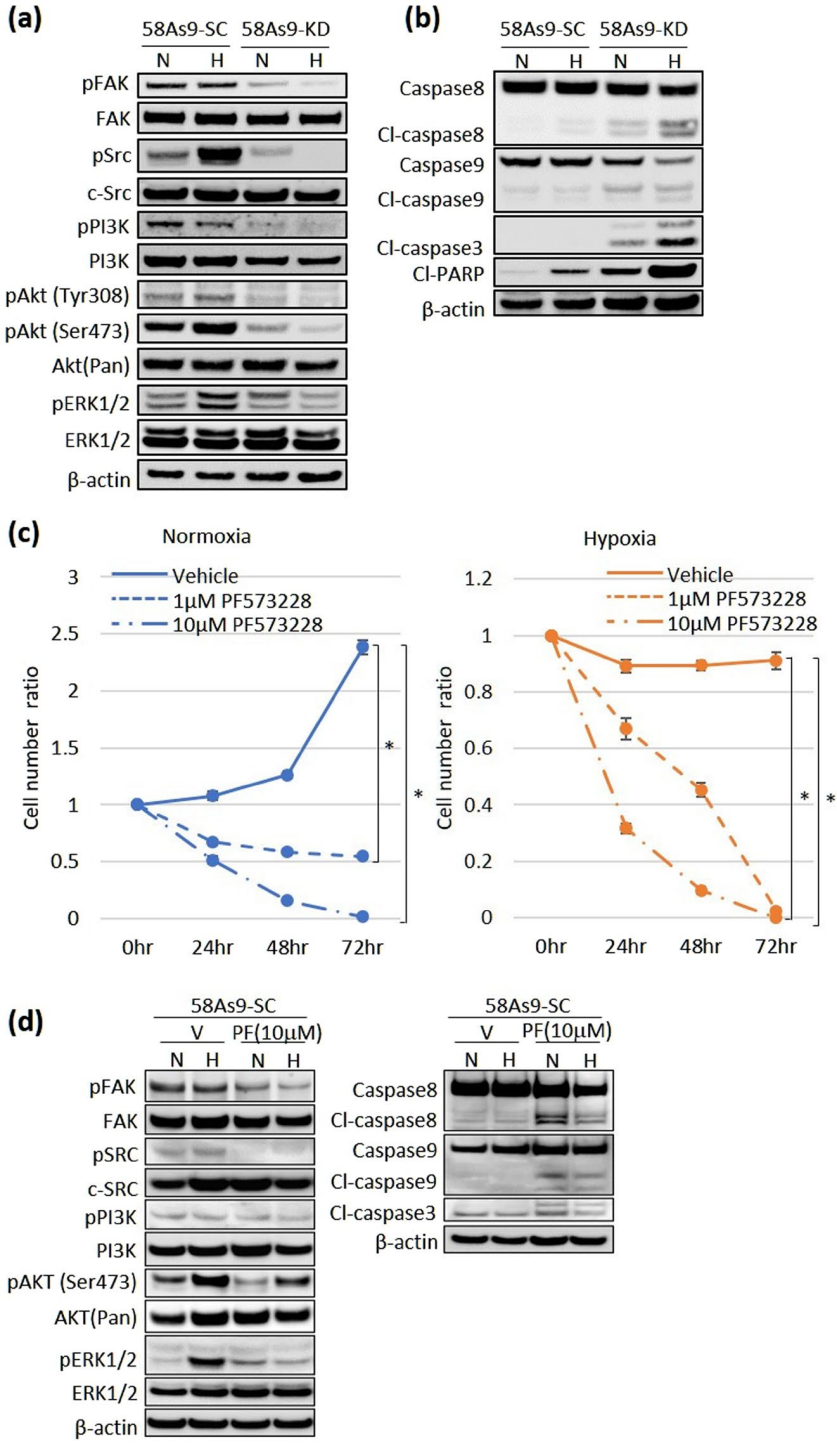
Analysis of the effect of ANGPTL4 knockdown on the expression of pro-survival signals and regulators of apoptosis. (**a**) WB analysis of the expression of pro-survival factors in 58As9-SC and 58As9-KD cells in suspension culture. pFAK, phosphorylated FAK; pSrc, phosphorylated Src; pPI3K, phosphorylated PI3K; pAkt (Tyr308), Akt phosphorylated on Tyr308; pAkt (Ser473), Akt phosphorylated on Ser473; pERK1/2, phosphorylated ERK1/2. N, normoxia; H, hypoxia. (**b**) WB analysis of the expression of regulators of apoptosis caspases-8, -9 and -3 in 58As9-SC and 58As9-KD cells. Cl-: cleaved form. (**c**) Cell number ratios of 58As9-SC cells, which were treated with the FAK inhibitor PF573228 at the indicated concentrations and times under normoxia (blue) or hypoxia (red). The significance of the difference of the cell number ratio between untreated and PF573228-treated-58As9-SC cells for 72 h was determined. P values ≤ 0.05 indicate a significant difference, as marked with an asterisk. (**d**) Left panel: WB analysis of the expression of pro-survival factors in 58As9-SC cells treated with or without PF573228 (PF) for 1 h. V: vehicle treatment. Right panel: WB analysis of the expression of apoptosis factors in 58As9-SC cells treated with or without PF573228 for 12 h. β-actin served as an internal control. The experiments were repeated three times. N, normoxia; H, hypoxia.

To assess how hypoxia-induced ANGPTL4 contributed to the resistance to anoikis of 58As9 SGC cells, we examined the effect of inhibiting ANGPTL4 expression on signalling through the pro-survival FAK/Src/PI3K-Akt/ERK pathway under normoxia and hypoxia (Fig. 7a). Phosphorylation of FAK, Src, PI3K, Akt and ERK was detected in 58As9-SC cells. Phosphorylation of Src, Akt (Ser473) and ERK1/2 was more strongly induced by hypoxia than by normoxia (Fig. 7a). In contrast, phosphorylation of FAK/PI3K/Akt/Src/ERK significantly decreased in 58As9-KD cells under both conditions (Fig. 7a). In particular, phosphorylation was more strongly suppressed under hypoxia than under normoxia (Fig. 7a).

We further investigated the activation of the regulators of apoptosis caspases-3, -8 and -9 as well as poly-ADP-ribose polymerase (PARP). Cleaved (Cl-) forms of caspases-3, -8, -9 and PARP were detected at higher levels in 58As9-KD cells than in 58As9-SC cells (Fig. 7b). Expression of these apoptotic regulators in 58As9-KD was more highly induced under hypoxia than under normoxia (Fig. 7b). To further confirm the pivotal role of FAK/Src/PI3K-Akt/ERK signalling in the resistance of 58As9 cells to anoikis, the effects of the FAK inhibitor PF573228 were evaluated in 58As9-SC cells. As shown in Fig. 7c, PF573228 (1 and 10 μM) treatment strongly decreased the cell number ratio with time in 58As9-SC cells under normoxia and hypoxia, and there was a significant difference in the cell number ratio at 72 h compared with that with vehicle (Fig. 7c). Moreover, PF573228 (10 μM) decreased the expression of pFAK, pSRC, pPI3K, pAKT (Ser473) and pERK1/2 in 58 As9-SC cells under normoxia and hypoxia (Fig. 7d). Conversely, the treatment increased the levels of Cl-caspases-8, -9 and -3 in these cells under normoxia and hypoxia (Fig. 7d).

### Effect of ANGPTL4 on peritoneal metastasis of xenografted GC cells

**Figure 8:**
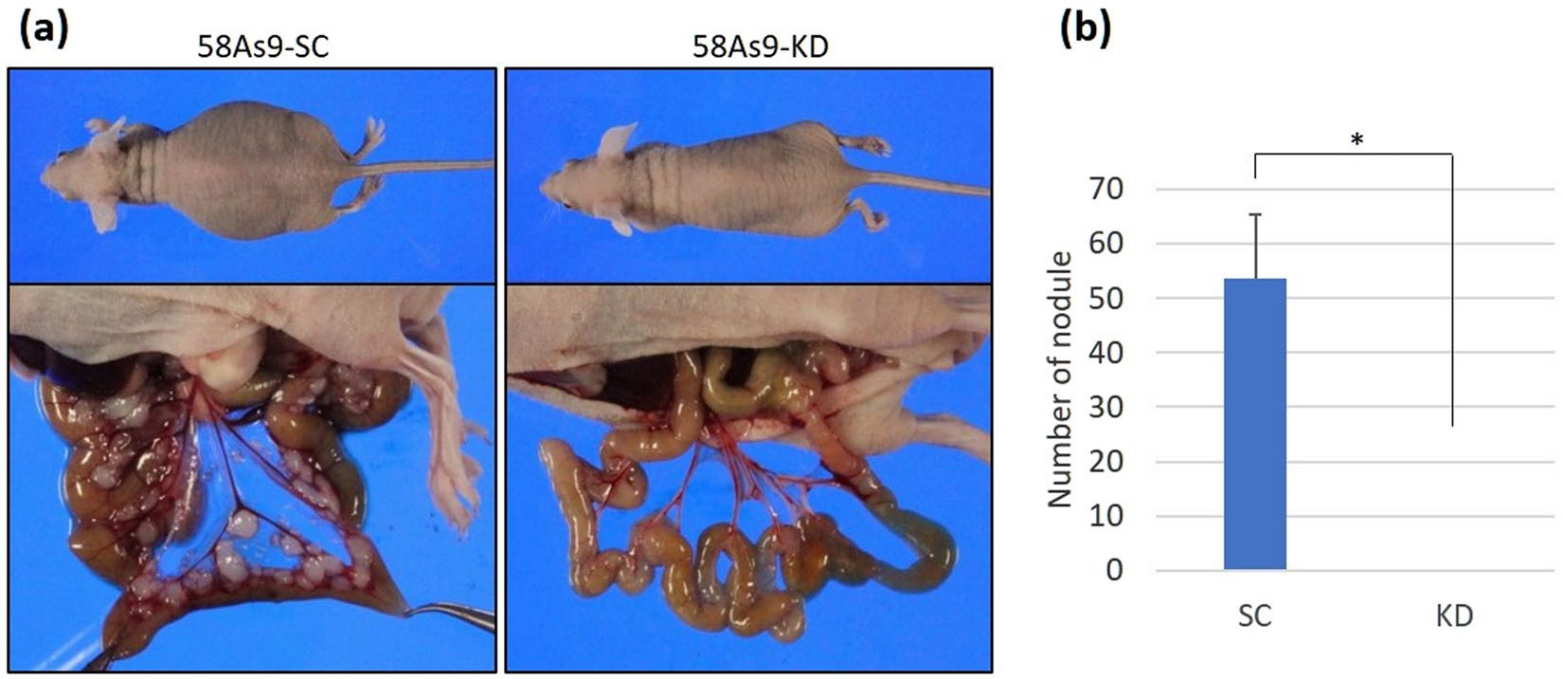
Effect of inhibiting ANGPTL4 expression on peritoneal dissemination of tumour cells. Nude mice were injected intraperitoneally (i.p.) with 4 × 10^6^ 58As9-SC (n = 5) or 58As9-KD (n = 5) cells. (**a**) Disseminated nodules and ascites formed in the five mice injected i.p. with 58As9-SC, but not in those with 58As9-KD cells. (**b**) The mean number of disseminated nodules was estimated in both 58As9-SC (SC) and 58As9-KD (KD) mice. P values ≤ 0.05 indicate a significant difference, as marked by an asterisk.

To investigate the effects of ANGPTL4 expression on resistance to anoikis, 58As9 transfectants were injected i.p. into nude mice and the development of peritoneal metastasis was evaluated. Massive ascites was present in all mice (n = 5) injected with 58As9-SC cells. Laparotomy revealed typical formation of peritoneal dissemination in 58As9-SC cells. In contrast, disseminated nodules or ascites fluid did not develop in mice (n = 5) injected with 58As9-KD cells (Fig. 8a). The mean number of nodules was estimated to be more than 50 in 58As9-SC mice, whereas there were no detectable nodules in 58As9-KD mice (Fig. 8b).

## Discussion

In the present study, we first evaluated the hypoxic induction of ANGPTL4 in nine GC cell lines, and revealed that hypoxia-induced ANGPTL4 expression was predominantly observed in 58As9 and 44As3, which are classified as SGC cells^22^. Upregulation of *ANGPTL4* mRNA under hypoxia is induced by HIF-1α^23–25^. Specifically, in hepatocellular carcinoma (HCC) cells, HIF-1α directly upregulates the transcription of *ANGPTL4* by binding to the HRE in the *ANGPTL4* promoter^25^. Therefore, we hypothesized that hypoxia-induced expression of *ANGPTL4* mRNA is regulated by HIF-1α in the SGC cell lines. In support of this hypothesis, we show here that HIF-1α predominantly regulated the hypoxic induction of *ANGPTL4* mRNA in 58As9 and 44As3 cells because siHIF-1α and siDK, but not siHIF-2α, significantly inhibited hypoxic induction.

Next, we established transfectants of 58As9 and 44As3 *ANGPTL4* KD cells. ELISA analysis demonstrated that ANGPTL4 secretion was completely suppressed in 58As9-KD cells under normoxia or hypoxia, whereas a partial blockade was observed in 44As3-KD cells. Thus, 58As9-SC and 58As9-KD cells were the focus of our experiments. The proliferation of 58As9-KD cells was significantly inhibited compared with that of 58As9-SC cells under normoxia or hypoxia, and cell cycle arrest at the G_1_ phase was strongly induced in hypoxic 58As9-KD cells. These results indicate that cell proliferation might be dependent on secreted ANGPTL4 in 58As9 cells under normoxia and hypoxia because ANGPTL4 secretion increased with time under both conditions in 58As9-SC cells. In particular, strong elevation of ANGPTL4 secretion by hypoxia may be required for escape from cell cycle arrest by 58As9 cells.

In contrast, the cell cycle data are consistent with our findings that the expression of the activator of proliferation c-Myc was significantly inhibited in normoxic and hypoxic 58As9-KD cells. In contrast, the cell cycle inhibitor p27 was conversely upregulated in KD cells and was expressed at higher levels in hypoxic cells than in normoxic ones. These results prompted us to explore TGF-β/Smad signalling in 58As9-KD cells because p27 induction following the inhibition of c-Myc expression is a target of TGF-β/Smad signalling that suppresses cell proliferation^26–30^. The demonstration here that the levels of pSmad2 and pSmad3 were increased in 58As9-KD cells, compared with those in 58As9-SC cells, provides compelling support for the contribution of TGF-β/Smad signalling to suppression of cell proliferation.

An ELISA also showed that TGF-β was more highly secreted from 58As9-KD than from 58As9-SC cells. These results imply that ANGPTL4 secreted from 58As9-SC cells may suppress TGF-β expression and may lead to the inactivation of Smad 2/3 signalling. Inhibition of the TGF-β/Smad pathway by ANGPTL4 may sustain the proliferation of 58As9-SC cells via downregulation of p27 and upregulation of c-Myc. To address the hypothesis, we treated 58As9-KD cells with the TGF-β inhibitor SB431542 and investigated whether inhibition of TGF-β/Smad signalling not only upregulated c-Myc but also downregulated p27 to reverse cell cycle arrest under hypoxia. We found that SB431542 (5 μM) completely blocked TGF-β/Smad signalling. SB431542 treatment weakly increased c-Myc expression in hypoxic 58As9-KD cells, whereas this treatment incompletely reduced p27 expression. However, the treatment did not affect cell proliferation or the cell cycle in hypoxic 58As9-KD cells. Thus, TGF-β/Smad signalling activated by ANGPTL4 KD did not fully solve the mechanism of cell cycle arrest in hypoxic 58As9-KD cells. Additional signalling pathways also affected by ANGPTL4 KD may be required to reverse the cell cycle arrest in hypoxic 58As9-KD cells. In addition, cell proliferation of 58As9-SC cells was inhibited by hypoxia compared with that under normoxia. The cell population in G_1_ phase was increased under hypoxia in these cells. Furthermore, p27 expression of 58As9-SC cells was modestly elevated by hypoxia. Therefore, hypoxic stimulation may decrease cell proliferation in 58As9-SC cells via p27 induction mediated by an ANGPTL4-independent mechanism.

We next employed a nude mouse xenograft model in an attempt to show that 58As9-KD cells completely lost tumorigenicity compared with 58As9-SC cells. The obtained findings suggest that a lack of ANGPTL4 expression in hypoxic 58As9-KD cells suppressed cell proliferation and that cell cycle arrest was strongly induced through downregulation of c-Myc and upregulation of p27. Hypoxia-induced cell cycle arrest may explain the inability of 58As9-KD cells to form tumours in nude mice. However, further study is necessary to fully elucidate the mechanism of cell cycle arrest induced by ANGPTL4 KD.

Anoikis is a form of apoptosis that occurs following the detachment of cells from extracellular matrix^31, 32^. Resistance to anoikis is a critical mediator of metastasis that enables cancer cells to survive during their invasion of distant organs^31, 32^. The histological characteristics of the SGC cell lines 58As9 and 44As3 analysed here reflect those of signet-ring cell carcinomas^22^. Further, the 58As9 and 44As3 cell lines were originally established from the ascites and pleural fluid of patients with SGC^22^. Therefore, we investigated whether ANGPTL4 is essential for the resistance of SGC cells to anoikis.

Under suspension culture, the proliferation of 58As9-SC cells was suppressed by hypoxia. In addition, the proliferation of 58As9-KD cells was significantly inhibited compared with that of 58As9-SC cells under normoxia or hypoxia. Flow cytometric analysis of suspension cultures of 58As9 cells demonstrated that apoptosis was more strongly induced in 58As9-KD cells than in 58As9-SC cells. The rate of apoptosis was also increased under hypoxia compared with that under normoxia. These results indicate that hypoxia-induced ANGPTL4 expression was critical for sustaining the resistance of SGC 58As9 cells to anoikis. In addition, it is speculated that hypoxia may inhibit cell proliferation in suspension-cultured 58As9-SC cells by increasing the cell population at the G_1_ phase, as observed in monolayer culture, because hypoxia modestly elevated apoptosis in these cells. To further test whether exogenous ANGPTL4 restored resistance to anoikis, 58As9-KD cells were treated with rF-, rN- and rC-ANGPTL4. rF- and rC-ANGPTL4, but not rN-ANGPTL4, significantly decreased the number of apoptotic 58As9-KD cells, and the inhibitory effect on apoptosis was more strongly induced under hypoxia than under normoxia. Thus, F- or C-ANGPTL4 secreted from hypoxic 58As9 cells may play an important role in the avoidance of anoikis via an autocrine loop mediated by the C-terminal fibrinogen-like domain^15, 16^. In the present study, treatment with rF- or rC-ANGPTL4 did not completely inhibit the anoikis of 58As9-KD cells. We therefore speculate that ANGPTL4 peptides produced from recombinant ANGTL4 cDNA, but not native forms, may not fully restore the resistance to anoikis of 58As9-KD cells.

Emerging evidence has revealed that specific integrins stimulate the downstream components of the FAK/Src pathway to transduce pro-survival signals to PI3K-Akt or ERK, leading to resistance to anoikis^23^. Moreover, tumour-derived ANGPTL4 interacts with integrins-β1 and -β5 to activate FAK, leading to the activation of Rac, which further activates the NADPH oxidase-dependent generation of O_2_^−^. The elevation of the level of O_2_^−^ subsequently activates Src, triggering the PI3K-Akt and ERK pro-survival pathways that can confer resistance to anoikis upon squamous carcinoma cells^23^.

Here, we show that the FAK/Src/PI3K-Akt/ERK pathway was activated in 58As9-SC cells more strongly under hypoxia than under normoxia. In contrast, pro-survival signals were drastically suppressed in hypoxic 58As9-KD cells. ANGPTL4/integrin primed-survival signalling, followed by activation of the FAK/Src/PI3K-Akt/ERK pathway, may play a pivotal role in conferring resistance to anoikis in hypoxic 58As9 cells.

When we investigated the activation of the regulators of apoptosis, we found that the intrinsic regulators caspases-9 and -3 as well as the extrinsic regulator caspase-8 were activated in 58As9-KD cells more strongly under hypoxia than under normoxia. Unique mechanisms of anoikis involve interactions between intrinsic and extrinsic apoptotic pathways, and the interaction of BAX with tBid plays a critical role^33–35^. Similar mechanisms might be induced during the anoikis of 58As9-KD cells. Together, these findings indicate a possible mechanism of hypoxia-induced ANGPTL4 expression that may strongly activate the FAK/Src/PI3K-Akt/ERK pro-survival pathway and subsequently avoid anoikis by suppressing the activities of regulators of apoptosis in 58As9 cells.

To further elucidate the ANGPTL4 primed resistance to anoikis, we investigated whether the FAK inhibitor PF573228 inhibited downstream signalling and induced anoikis in 58As9-SC cells. The results clearly show that the time- and concentration-dependent effects of PF573228 suppressed cell proliferation in 58As9-SC cells, with stronger inhibition under hypoxia than under normoxia. Moreover, PF573228 inactivated downstream Src/PI3K-Akt/ERK signalling and conversely activated apoptotic factors including caspases-3, -8 and -9. Taking these findings together, the ANGPTL4/integrin interaction may activate FAK on the surface of 58As9 cells to transduce survival signals to Src/PI3K-Akt/ERK, and subsequently suppress the apoptotic pathway.

The acquisition of resistance to anoikis plays an important role in the development of peritoneal dissemination in patients with GC or ovarian cancer^36, 37^. When we used a nude mouse xenograft mode to investigate the role of ANGPTL4 in peritoneal metastasis, we found that peritoneal dissemination with ascites fluid was present in mice injected with 58As9-SC cells. In contrast, disseminated nodules and ascites were not observed in mice engrafted with 58As9-KD cells. These findings strongly suggest that ANGPTL4 expression is critical for promoting the resistance of SGC cells to anoikis.

In conclusion, the present study demonstrates the essential roles of ANGPTL4 in the progression of SGC. A hypoxic environment may induce HIF-1α in the SGC tumour, and HIF-1α-induced ANGPTL4 may promote tumour growth by upregulation of c-Myc and downregulation of p27. As SGC progresses, the cancer cells may acquire resistance to anoikis through ANGPTL4-dependent activation of FAK/Src/PI3K-Akt/ERK signalling, leading to the development of peritoneal metastasis. Treatment with a specific ANGPTL4 inhibitor such as a neutralizing antibody may suppress tumour growth and peritoneal metastasis in patients with SGC.

## Methods

### Cell culture

The human GC cell lines 58As9, 44As3, HSC45, HSC57, MKN1, MKN7, MKN45, MKN74 and KATO-III were investigated. Dr. Yanagihara (National Cancer Center Hospital, Kashiwa, Japan) kindly provided the 58As9, 44As3, HSC45 and HSC57 cell lines. We purchased MKN1, MKN7, MKN45, MKN74 and KATO-III from RIKEN Cell Bank (Tsukuba, Japan). The characteristics of cell lines and their origins are as follows: 58As9 and HSC45, signet-ring cell carcinoma, malignant ascites; 44As3, signet-ring cell, pleural effusion; HSC57, signet-ring cell/well-differentiated adenocarcinoma, pleural effusion; MKN1, adenosquamous carcinoma cell, lymph node metastasis; MKN7, well-differentiated adenocarcinoma, lymph node metastasis; MKN45, poorly differentiated adenocarcinoma, liver metastasis; MKN74, moderately differentiated adenocarcinoma, liver metastasis; and KATO-III, signet-ring cell, stomach. All GC cell lines were grown in RPMI-1640 medium (Sigma-Aldrich, St. Louis, MO, USA) containing 10% foetal bovine serum (Sigma-Aldrich) and 100 μg/mL kanamycin (Meiji, Tokyo, Japan) at 3°C in a humidified atmosphere. Cells were cultured under normoxic (5% CO_2_ in air) and hypoxic (1% O_2_ and 5% CO_2_ in N_2_) conditions.

### Reagents

Full-length (F-ANGPTL4), and N-terminal (N-ANGPTL4) and C-terminal (C-ANGPTL4) recombinant proteins were purchased from R&D Systems (Minneapolis, MA, USA). The TGF-β inhibitor SB431542 was purchased from Sigma-Aldrich and the FAK inhibitor PF573228 was purchased from Santa Cruz Biotechnology, Inc. (Dallas, TX, USA).

### RNA interference

Oligonucleotides encoding siRNAs targeting HIF-1α or HIF-2α were used to transfect 44As3 and 58As9 cells. The target sequences of the siRNAs are as follows: siHIF-1α, 5′-CAAAGUUCACCUGAGCCU-3′ and siHIF-2α, 5′-GCAAAUGUACCCAAUGAU-3′. The MISSION siRNA Universal Negative Control (Sigma-Aldrich) served as a control. Transfection was performed using a MicroPorator-mini (Digital Bio Technology, Seoul, South Korea), in accordance with the manufacturer’s instructions.

### Establishment of KD cells that stably inhibit ANGPTL4 expression

The pBAsi-hU6 Pur plasmid vectors (Takara Bio, Kusatsu, Japan) encoding an ANGPTL4-shRNA as well as a control Scramble shRNA were constructed. The target sequences are as follows: ANGPTL4, 5′-GAAACTTGTGGACAGAGAA-3′, and Scramble, 5′-TCTTAATCGCGTATAAGGC-3′. We used the electroporator described above to transfect 44As3 and 58As9 cells with these plasmid vectors. KD cells that stably suppress ANGPTL4 expression and control cells (44As3-SC or 58As9-SC) were selected using puromycin. We picked up and expanded puromycin-resistant colonies. Transfectants with the lowest levels of ANGPTL4 were designated 44As3-KD and 58As9-KD.

### Protein extraction and WB analysis

Extraction of proteins from whole-cell lysates was performed as described previously^12, 13^. Extraction of proteins from conditioned culture medium was performed as follows: Cells (5 × 10^6^) were cultured in RPMI-1640 medium without fetal bovine serum (FBS) for 24 h. Conditioned medium was harvested and added to trichloroacetic acid at 20% of the final concentration. This sample was incubated on ice for 20 min and centrifuged at 15,000 × *g* for 5 min. The pellet was washed in cold acetone and then dissolved in lysis buffer containing a protease inhibitor cocktail (Roche, Mannheim, Germany).

WB analysis was performed as previously described^12, 13^. Briefly, 30 μg of protein was transferred onto the membrane. The membrane was incubated with primary antibodies overnight at 4 °C and then with secondary antibodies for 30 min. Immune complexes were detected using ECL Plus (GE Healthcare, Piscataway, NJ, USA) and an LAS-3000 (GE Healthcare). β-actin served as an internal control.

### Antibodies

The primary antibodies (and their final dilutions) used in this study were as follows: ANGPTL4 (goat polyclonal, immunogen: 26–406 a.a., 1:2000; R&D Systems, Minneapolis, MN, USA), Ab2: ANGPTL4 (mouse polyclonal, immunogen: 26–406 a.a., 1:1000; Abnova, Taipei, Taiwan), HIF-1α (1:1000; Becton-Dickinson Biosciences, Franklin Lakes, NJ, USA); HIF-2α (1:1000; Santa Cruz Biotechnology, Inc.); caspase-8, caspase-9, cleaved caspase-3, cleaved PARP, p-Smad2, Smad2, p-Smad3, Smad3, Smad4, FAK, p-PI3K, p-Akt (Thr308), p-Akt (Ser473), Akt(pan), p-Src, Src, p-ERK1/2, ERK1/2, c-Myc, p27kip1 (1:1000; Cell Signalling Technology, Danvers, MA, USA); p-FAK, PI3K (1:500; Proteintech, Chicago, IL, USA); and β-actin (1:10,000; Sigma-Aldrich). The HRP-conjugated secondary antibodies were as follows: goat anti-mouse IgG, goat anti-rabbit IgG and donkey anti-goat IgG (1:2000 each; Santa Cruz Biotechnology, Inc.). ANGPTL4 or TGF-β secreted into the culture supernatant was evaluated using specific ELISA kits (R&D Systems, Minneapolis, MN, USA), in accordance with the manufacturer’s instructions.

### Extraction of RNA and quantitative real-time polymerase chain reaction (RT-qPCR) analysis

Total RNA was extracted as previously described^12, 13^. RT-qPCR was performed using the CFX Connect Real-Time PCR Detection System (Bio-Rad, Philadelphia, PA, USA) with SsoAdvanced Universal SYBR Green Supermix (Bio-Rad), in accordance with the manufacturer’s protocol. The qPCR program was performed using two StepAmp procedures, as described previously^13^. *ACTB* mRNA served as an internal control. Primers for *ANGPTL4* and *ACTB* were as follows: *ANGPTL4* (sense, 5′-TCCGTACCCTTCTCCACTTG-3′; antisense, 5′-AGTACTGGCCGTTGAGGTTG-3′) and *β-actin* (sense, 5′-ACGCCTCTGGCCGTACCACT-3′; antisense, 5′-TAATGTCACGCACGATTTCCC-3′).

### Cell proliferation assay

Cell proliferation was assessed by counting cells. Briefly, 44As3 (5.0 × 10^4^) or 58As9 (1.5 × 10^5^) cells were incubated under normoxia or hypoxia for 24, 48 and 72 h. The cells were trypsinized and counted using a light microscope.

### Anchorage-independent cell viability (anoikis) assa

Ultra-low-attachment culture plates (Corning Inc., NY, USA) were seeded with 44As3 (5.0 × 10^4^) or 58As9 (1.5 × 10^5^) cells and incubated under normoxia or hypoxia for 24, 48 and 72 h. Suspended cells were collected and counted.

### Cell cycle analysis

The cell cycle was analysed using flow cytometry with propidium iodide (PI). Briefly, 3 × 10^5^ of each of 58As9-SC or 58As9-KD cells were cultured under normoxia or hypoxia for 24 h, harvested, fixed in 70% cold ethanol and then treated with ribonuclease (0.25 mg/ml) and PI (50 µl/ml). The samples were analysed using a FACSVerse flow cytometer (Becton-Dickinson Biosciences) and FlowJo ver. 7.6 software (FlowJo LLC, Ashland, OR, USA).

### Flow cytometric detection of apoptotic cells

Apoptosis was analysed using flow cytometry with an Annexin V-FITC Apoptosis Detection Kit (BioVision, Milpitas, CA, USA). Briefly, 58As9-SC and 58As9-KD cells were cultured in ultra-low-attachment vessels, harvested and resuspended in 1 × Binding buffer, and then incubated with Annexin V-FITC and PI. Annexin V-FITC and PI signals were assessed using FACSVerse. Cell populations were defined as follows: Annexin V (−)/PI (−), viable; Annexin V (+)/PI (−), early apoptotic; Annexin V (+)/PI (+), late apoptotic; and Annexin V (−)/PI (+), necrotic or damaged.

### Mouse studies

Five-week-old female BALB/c nude mice were obtained from CLEA Japan (Tokyo, Japan). The Animal Care Committee of Saga University approved the experiments using mice. All methods were performed in accordance with the relevant guidelines and regulations.

### Subcutaneous xenograft mouse models

The flanks of nude mice (n = 3) were injected subcutaneously with 3 × 10^6^ 58As9-SC or 58As9-KD cells, and tumour size was measured 5 days later. Tumour volume was calculated as follows: (L × W^2^)/2 mm^3^, where L and W are the long and short axes of the tumour, respectively. Mice with tumours >10 mm were sacrificed using an overdose of general anaesthesia.

### Peritoneal dissemination of xenografts

Nude mice were injected i.p. with 58As9 transfectants. Each of five mice was injected with 4 × 10^6^ of 58As9-SC or 58As9-KD cells. All mice were sacrificed on day 18 after i.p. injection and underwent laparotomy to evaluate peritoneal dissemination.

### Statistical analysis

*In vitro* studies were performed in triplicate and repeated three times. Three (six tumours per transfectant) and five mice per transfectant were implanted subcutaneously or i.p., respectively. Data are expressed as the mean ± standard deviation (SD), and analyses were performed using SPSS 22 software (SPSS Inc., Chicago, IL, USA). Student’s *t* test and the Mann–Whitney test were used to compare two groups, and P < 0.05 was considered to indicate a statistically significant difference.

## Electronic supplementary material

Supplemental Figures
