# Supplementary material for: Hypoxia-induced ANGPTL4 sustains tumour growth and anoikis resistance through different mechanisms in scirrhous gastric cancer cell lines
Source: Sci Rep. 2017 Sep 11;7:11127. doi: 10.1038/s41598-017-11769-x (PMC5594024; doi:10.1038/s41598-017-11769-x)
Supplement: Supplementary file 1 — Supplemental Figures [file 41598_2017_11769_MOESM1_ESM.pdf]

Supplemental information

Hypoxia-induced ANGPTL4 sustains tumour growth and anoikis resistance through different mechanisms in scirrhous gastric cancer cell lines

Koichi Baba[1], Yoshihiko Kitajima*[1,2], Shuusuke Miyake[1], Jun Nakamura[1], Kota Wakiyama[1], Hirofumi Sato[1], Keiichiro Okuyama[1], Hiroshi Kitagawa[1], Tomokazu Tanaka[1], Masatsugu Hiraki[3], Kazuyoshi Yanagihara[4] & Hirokazu Noshiro[1]

[1]Department of Surgery, Saga University Faculty of Medicine, 5-1-1, Nabeshima, Saga-shi, Saga 849-8501, Japan

[2]Department of Surgery, National Hospital Organization Higashisaga Hospital, 7324, Ooaza Harakoga, Miyaki-cho, Miyaki-gun, Saga 849-0101, Japan

[3]Department of Surgery, Saga-ken Medical Centre Koseikan, 400, Ooaza Nakahara, Kase-machi, Saga-shi, Saga 840-8571, Japan

[4]Division of Translational Research, Exploratory Oncology Research & Clinical Trial Center, National Cancer Center, 6-5-1 Kashiwanoha, Kashiwa-shi, Chiba 277-8577, Japan

*Corresponding author

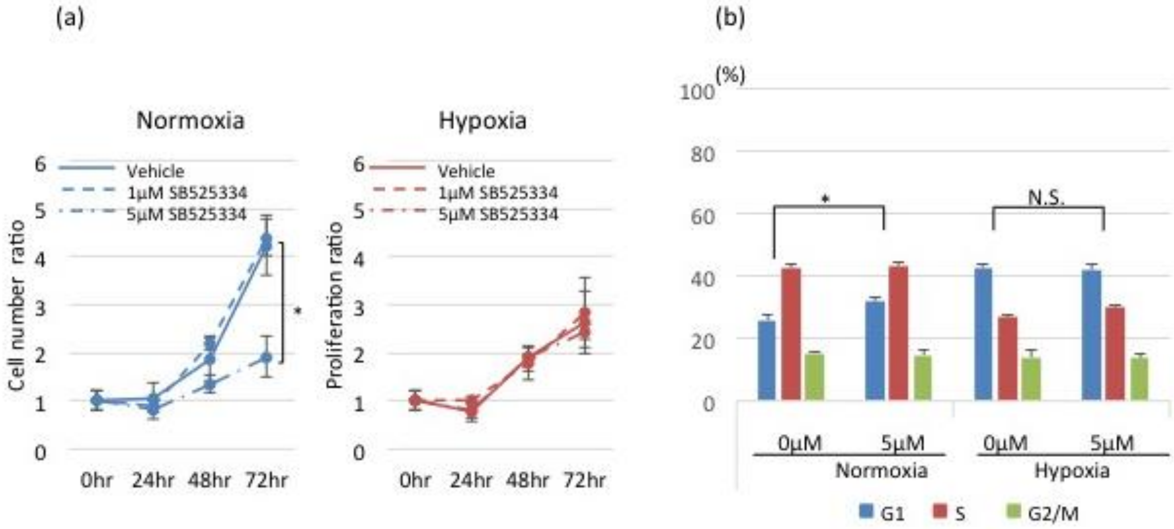

Figure S1. The effect of the TGF-β inhibitor SB431542 on the cell cycle in 58As9-KD cells.

(A) 58As9-KD cells were treated with or without SB431542 (1μM, 5 μM) at the indicated times under normoxia or hypoxia, and the cell number ratio was estimated. (B) 58As9-KD cells were treated with or without SB431542 (5 μM), and cultured for 24 hr under normoxia and hypoxia. The proportions of cells in the G1, S or G2/M phase of the cell cycle are shown. P values ≤ 0.05 indicate a significant difference and are marked with an asterisk.

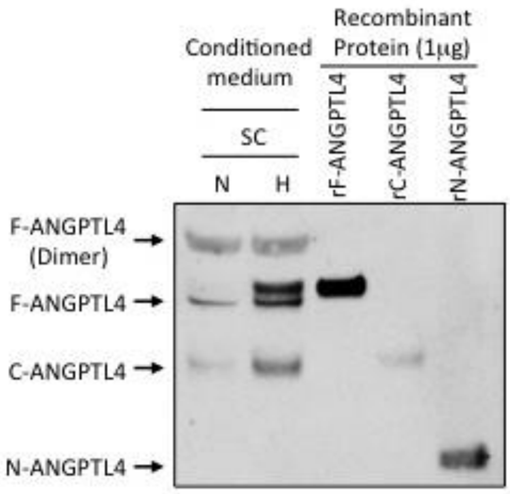

Figure S2. WB analysis of F-, N- and C-ANGPTL4 in culture supernatants from 58As9-SC cells using the ANGPTL4 antibody Ab2.

In parallel, three forms of recombinant (r) peptides (1 μg) were subjected to WB. F- and C-ANGPTL4, but not N-ANGPTL4, was detected in culture supernatants of 58As9-SC cells under both normoxia (N) and hypoxia (H), and the intensities were stronger in hypoxia compared with those in normoxia. rF-ANGPTL4 and rN-ANGPTL4 peptides were clearly detected by Ab2, whereas the rC-ANGPTL4 band was weakly detected.
